# Apoplexy

**Published:** 1854-11

**Authors:** 


*Apoplexy.*—Dr. Stone, of New Orleans, publishes some rambling remarks upon this and kindred diseases, in the Medical News of that city, in which there are some valuable practical ideas. He says there has been an unusual number of deaths during the past year, from nervous diseases; such as apoplexy, epilepsy, paralysis tetanus, convulsions in children, &c. He thinks an epidemic influence of a peculiar diathesis must prevail, to account for the prevalence of these, as of other diseases. A state of nervous debility or exhaustion, he considers the prominent feature, and that the fluids play a secondary part, whether consdered in quantity or distribution. As in other cases which occasionally become epidemic, all the local and exciting causes that are known, may fail to produce them at other times. Apoplexy he considers a nervous disease, and refers to the erroneous notice so prevalent, that there is an undue rush of blood to the brain, which, being encased in the unyielding scull, cannot contain more at one time than another. If there be an effusion of blood or serum, it must be at the expense of the circulating fluids. The interrupted respiration in apoplexy checks the return of venous blood from the brain, and causes a proportional exclusion of arterial blood, and thus the effect may be mistaken for the cause. Persons with large heads and short necks are not on this account more prone to brain disease, except as their propensities and habits of life lead to it, the nervous system being brought to such a state of exhaustion as to overtax its energies to manage an overloaded stomach. Persons with small heads and long necks, find their nervous systems give way in some other parts before the apoplectic state is brought about. When this is not the case, they have apoplexy like others. Excessive indulgences do not show externally in such subjects, but tell upon tha brain with equal certainty as in those of plethoric habits

He deems blood-letting on improper remedy, although temporary benefit may be derived from it in cases of great pletnora A peculiar state of the stomach, independent of indigestible food, exercises a depressing influence on the brain, which blood-letting does not relieve. Cathartics which act without exciting the secretions afford little relief, but mercurial purgatives seldom fail, being aided by small doses of saline laxat ves. He makes less account of emetics than one would be led to expect from a knowledge of his pathology; andless than we are disposed to do, from experience in the treatment of such cases. Vomiting, however produced, we have found the most efficient of all remedies in these diseaes, whether in adults or c hildren. Titilation of the throat with a feather will often excite, emesis and afford prompt relief, when it is impossible to administer remedies of any kind. He advises croton oil for the convenience of exhibition, and stimulating injection, and carbonate of ammonia as a stimulant and thinks the rectum absorbs as readily in such cases as if nothing was the matter. Dr. S. believes that all functional disorders of the nervous system are disposed to become periodic, and tend to a favorable issue if interrupted respiration, and engorgements of the lungs and brain can be warded off; but it must be borne in mind that his observations are made in New Orleans, where periodicity is the prominent characteristic of diseased conditions.

